# Resilience Capability and Capacity in Unexpected Crises: Experiences and Lessons Learned in a Healthcare Organisation during the COVID-19 Pandemic

**DOI:** 10.1155/2023/6418267

**Published:** 2023-05-23

**Authors:** Nanna Gillberg, Linda Ahlstrom, Annette Erichsen Andersson, Sara L. Fallman, Alessio Degl'Innocenti, Ingibjörg H. Jonsdottir, Helle Wijk, Ewa Wikström

**Affiliations:** ^1^The School of Business, Economics and Law, Department of Business Administration, University of Gothenburg, Gothenburg, Sweden; ^2^The Sahlgrenska Academy, Health and Care Sciences, University of Gothenburg, Gothenburg, Sweden; ^3^Department of Orthopaedics, Region Västra Götaland, Sahlgrenska University Hospital, Gothenburg, Sweden; ^4^Department of Work Life and Social Welfare, University of Borås, Borås, Sweden; ^5^Centre for Ethics, Law and Mental Health (CELAM), Department of Psychiatry and Neurochemistry, Institute of Neuroscience and Physiology, Sahlgrenska Academy, University of Gothenburg, Gothenburg, Sweden; ^6^Region Västra Götaland, University of Gothenburg, Regionhälsan, Gothenburg, Sweden; ^7^Region Västra Götaland, Institute of Stress Medicine, University of Gothenburg, Gothenburg, Sweden; ^8^School of Public Health and Community Medicine, Institute of Medicine, Sahlgrenska Academy, University of Gothenburg, Gothenburg, Sweden

## Abstract

**Aim:**

The current article aims to gain insight into (a) what characterises organisational resilience during an unexpected crisis such as COVID-19 and (b) how organisations respond to developments in their environments.

**Background:**

In times of societal crises, such as the COVID-19 pandemic, the resilience of the healthcare organisation is tested.

**Method:**

This research is based on a case study in a university hospital and a county hospital in Sweden using surveys with both structured and open answers.

**Results:**

The result shows ambiguity and “polarised” experiences, emphasising flexibility vs. structure, clear hierarchical information vs. spaces for peer learning through dialogue, and focus on acute care vs. determination to continue with core operations.

**Conclusion:**

The article concludes that the pandemic resulted in paradoxes, tensions, and new experiences in organisational processes and interactions. These create opportunities for learning not only during crises but also for improving nursing management in both acute and planned care. Three relations are important in building organisational resilience in crises: resilience capability, resilience capacity, and sustainable resilience practices. *Implications for Nursing Management*. Organisational resilience under extraordinary circumstances, such as a pandemic, as well as enhancing the previous literature on nursing management that offer a more individually oriented perspective.

## 1. Introduction

In times of societal crises, such as the COVID-19 pandemic, the resilience of the healthcare organisation is tested for the unpredictable. Organisational resilience can support the resilience of work at the organisational level, through preparedness for crises and extraordinary events, improvising with existing resources to develop solutions for unexpected situations and problems [1, 2]. Resilience can be defined as the positive adaptation to significant adversity [3]. It can serve to mitigate negative outcomes such as burnout and anxiety during a pandemic as well as improve patients' care [4]. Labrague and Santos [5] define personal resilience as a person's capacity to recover from a stressful event or to cope with or manage heavy workload and stress during a crisis or disaster event. For nursing management, resilience is a pivotal issue to nursing administrators and managers both with regards to recruiting and retaining nurses, i.e., to both competence supply and staff retention [6, 7] and to avoid burnout and work-related negative health effects [8].

Resilience must be supported by leadership strategies within the healthcare sector that contribute to creating supportive work environments [9]. Organisational support is defined as structural support, efficient communication, provision of a safe work environment, trainings related to COVID-19, and monitoring of healthcare workers' (HCWs) health and well-being. But resilience at the individual level has a limited effect, unless the work of strengthening resilience also permeates the team and organisational levels [10]. Rangachari and Woods [1] emphasise the importance of seeing organisational resilience as a system developed by the organisation and managers to learn from the work of individual employees, with a focus on proactivity, adaptation, and recovery.

Furthermore, Duchek [11] highlights a capability-based conceptualisation of organisational resilience in response to a crisis as three stages. The proactive stage occurs before an unexpected event and is defined as anticipation through observation, identification, and preparation to have resources available. The concurrent action stage occurs during the unexpected event through accepting, developing, and implementing solutions to support and use the social resources efficiently. The reactive action stage occurs after an unexpected event through reflection, learning, and change in which power and responsibility structures become central. In this study, the framework on organisational resilience that combines a processual approach and a focus on resilience capabilities, suggested by Duchek, will be used.

The mental well-being of nurses during COVID-19 has been stated to constitute a key component in the securing and rebuilding of the healthcare workforce following the pandemic [12]. Several studies have focused on how nurse leaders can support nurse staff and encourage a supportive work environment that mitigates stress and experiences of insecurity and anxiety at work [9, 13, 14]. Other studies have highlighted the role of nurse managers in creating such environments and how the responsibility it entails can lead to stress and burnout [15, 16]. Against this background, these studies stress the importance of research focusing nurse managers' well-being and resilience.

The importance of implementing resilience-promoting measures on an organisational level to manage a crisis is highlighted [10]. Such measures include balancing structures, such as a learning organisation. When organisational support is in place, it also promotes resilience by allowing hierarchies to be bypassed, micromanagement to be eliminated, and innovative approaches to focus on core patient-related activities [17]. The ability to predict the impact of severe crises as well as restore safe conditions increases by learning from previous crises and by adapting the organisation to new circumstances [18]. The importance of organisational conditions to support resilience is highlighted by Sihvola et al. [19], who emphasise a relational leadership style, supportive and safe work environment, and adequate communication to support nurses' resilience.

The following aspects have been highlighted as pivotal components for a supportive work environment that supports mental well-being: empathy in leadership and communication with staff [20–23], provision of appropriate resources [24], gaining access to sufficient information [25, 26], and functioning communication and empowerment of staff to take action [27]. Healthcare workers in a Swedish study, working with COVID-19-infected patients, reported factors such as worries about being infected, having to change workplace, and lack of protective equipment affecting their work capacity negatively [28]. Furthermore, another study using the same data set showed that psychiatric nurses were the most affected among HCWs in psychiatric clinics although psychiatric services did not directly care for patients with severe COVID-19 infection [29].

Most studies on resilience among first-line healthcare environments during COVID-19 apply an individual perspective, focusing on individual traits and leadership styles [12, 30, 31]. Scholars, however, have begun to challenge the focus on resilience as an individual ability and responsibility, arguing for a view of it as a joint responsibility of the individual and the organisation [16].

Accordingly, more detailed knowledge is needed on organisational resilience within the area of nursing management [6, 32]. The aim of this study is to increase the understanding of complex resilience capabilities by studying processes and actions during an unexpected pandemic crisis. With a focus on how organisations respond to and manage developments in their environments in the face of a crisis, the study aims to provide insight into effective nursing management of resilience in practice.

Research question: What are the characteristics of the experiences, structures, and processes that promote organisational resilience among employees and managers?

## 2. Methods

### 2.1. Setting

The study was conducted at two hospitals in Sweden, a university hospital and a county hospital in Västra Götaland region. All hospital administrations in the region were invited to participate, of which one university hospital and one county hospital responded positively to the opportunity. During the pandemic, the hospitals provided frontline care to COVID-19 patients. The university hospital with around 17,000 employees is one of the largest in Sweden, focusing on innovation and clinical research, with a broad cooperation with academia, industry, and patients. They have leading expertise in around 25 specialist areas, including cardiovascular care, reconstructive surgery, children's care, vaccinations, immunology, rheumatology, and catheter interventions, thus caring for patients from all over the country. The county hospital with a couple of thousand employees provides care for inhabitants equivalent to a small city. The hospital offers high quality care in all specialties, has extensive experience in working with process-oriented improvements in healthcare, and also has a research department with a university hospital setting.

This qualitative study is a part of a larger research project, with the aim of investigating the work environment and health of employees at a university hospital and a county hospital during a pandemic in a region in the western part of Sweden. The study included a total of 7740 respondents who were willing to participate in the research project. Detailed information on the questionnaire, and inclusion and exclusion criteria can be found in the article by Jonsdottir et al. [28]. It is based on 4097 (53%) respondents' free-text answers from managers and employees who completed the questionnaire and chose to comment on the included open-ended questions. The web-based questionnaire was administered in collaboration with the hospitals' Human Resources departments and distributed to all hospital employees (20314) eligible for study participation. The questionnaire invitation was sent by email in the first week of September 2020, with a link to an anonymous survey, and was followed up by a reminder email. Individuals could respond within 35 days [28]. The questionnaire included a total of 22 questions with subquestions and including three open-ended questions. The questionnaire took 10–15 minutes to complete. The questionnaire included questions pertaining to positive and negative experiences during the spring of 2020 when the COVID-19 pandemic was at its peak. Managers were asked questions about what organisational conditions had been important to them in their work as a manager in the spring of 2020 (e.g., clarity in decisions and procedures, division of responsibilities, resources, support, participation, and scope for action) and what organisational conditions they found were lacking in their work as a manager during the same period. The sample distributed by gender, age, and professional role is depicted in Table 1.

The data constitute experience-based knowledge. A central part of the study is to identify processes and factors, which are crucial for organisational resilience and for organisational conditions necessary for organisational learning that can function as a balancing structure. By mapping processes and factors that affect resilience, it is possible to create new knowledge that can be translated into clinical work to ensure adequate crisis preparedness in healthcare organisations and enable improved ways of working with acute care and planned care under regular circumstances.

The study was approved by the Swedish Ethical Review Authority (ref. 2020-04771), and participants provided informed consent. The study was conducted in compliance with the Helsinki Declaration.

### 2.2. Analysis of Free-Text Responses in the Survey

**Figure 1:**
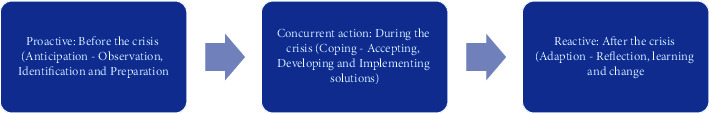
A capability-based conceptualisation of organisational resilience [11].

In the analysis, we draw on the Duchek [11] framework on organisational resilience and show how processes and practices relate to how the studied organisation responded to developments in its environment when facing a crisis.  Figure 1 summarises the central stages and processes suggested by Duchek [11] (p. 224).

The analysis was an iterative process of interpretation and sense making that began with initial statements in the free-text responses. The first reading and the initial categorisation of statements in the free-text responses were marked using patterns and links in their descriptive content. The next step was done based on the chosen theoretical framework in search of themes to categorise organisational resilience and how the organisation responded to developments in their environments in the face of a crisis. Finally, an iterative analysis was performed of the data encoded in these categories, with examples and illustrative quotes of organisational resilience that combine a processual approach and a focus on resilience capabilities suggested by Duchek (see Figure 1).

## 3. Results

The findings revealed four important processual practices constructing organisational resilience capability during the crisis at a university hospital and a county hospital caring for COVID-19 patients: (1) problem-solving orientation; (2) cooperation, peer support, and trust; (3) organisational learning and knowledge acquisition; and (4) information and communication. Questionnaire statements from each theme are presented in Table 2 and in the subsequent thematic presentation of our results.

An analysis of the four processual practices from the conceptual framework suggested by Duchek [11] shows that the three different temporal stages merge, while the prioritised focus in the conceptualising of *organisational resilience capability* is on concurrent action and the ongoing dynamic between the four processual practices. Furthermore, the other two stages (the proactive stage and the reactive stage) become more important to conceptualise *organisational resilience capacity* building practices, i.e., what practices are of importance for preparing, reflecting, learning, and changing before and after a crisis.

The result shows a pattern of ambiguity and “polarised” experiences at the operational level, emphasising the importance of flexibility vs. structure, clear hierarchical information vs. spaces for peer learning through dialogue, focus on acute care vs. a determination to continue with core operations.

### 3.1. Problem-Solving Orientation

A recurring theme in the survey responses was how the crisis brought a problem-solving orientation to the forefront, while a focus on policies and organisational charts was pushed aside. Decisions were made on the floor, mostly concerning the core work of care, i.e., patient-related matters. Time was no longer exceedingly spent on administration, HR, finance, and reports. Instead, patient work took the centre stage. This supported the organisation.

In many statements, an action-oriented approach was articulated to speed up processes, favouring a slower and less efficient decision-making process where issues are discussed broadly and anchored within the organisation.

The strength of focusing on a common goal and getting things done in an otherwise slow organisation (administrator, e.g., economist, HR specialist, communicator, and IT administrator).

Collaboration transcending organisational and disciplinary boundaries was set against conventional organising, abiding by set boundaries and organisational schemes.

To stop looking at organisational units and look at the whole, and that we all help each other, has been fantastic (First-line manager).

Furthermore, a focus on core activities, such as prioritising caring for patients instead of meetings and other less pressing issues, can be seen as an outcome of the action orientation. This prioritisation contributed to the resilience of the organisation by ensuring that the patients were referred to the right service.

The right patients came to specialist care. Primary care patients were properly managed in primary care. More time for patient work, less time for appointments. It felt very good. Unfortunately, we are now seeing a return to more meeting time and less understanding from clinic management to focus on patients (Specialist doctor).

Many comments testified to an increased focus on core activities and that pressing matters were prioritised over presumably less important issues.

When the pandemic hit, hospital leaders, managers, and administrators/care developers suddenly agreed that it is healthcare we should be producing, instead of gossipy printed materials, exciting patient-oriented projects, improvement boards, X-matrices, and other drivel. Suddenly, the focus was on taking care of as many patients as possible and the money was rolling in (Specialist doctor).

### 3.2. Cooperation, Peer Support, and Trust

Also prominent in the survey responses was the perception that collaboration between clinicians had increased during COVID-19. Many respondents expressed that during the crisis, the organisation had become more collective, deviating from the individual focus that under more general conditions seemed to dominate. In the observed case, decent work, organisational conditions, and organisational resilience were facilitated by a sense of community, collaboration, and collective efforts. The interrelatedness between the organisational levels was mainly depicted as enacted between organisational units and horizontal rather than vertical.

Positive That the Cooperation between the Different Units Has Been So Good (Specialist Doctor)

Managers viewed the increased cooperation between colleagues and professional groups within and between units as a positive aspect of the crisis. Both nurses and physicians expressed that working during the crisis has increased community and cohesion among staff.

The positive thing has been the sense of community with colleagues and that everyone did their best. You felt you were making a big contribution (Assistant nurse).

The sense of community was further emphasised as important for those employees who were moved between units, depending on needs and labour shortages.

You felt very welcome at your “new” workplace, and everyone was very grateful for your help. It was easy to feel like “one of the bunch,” and everyone understood that you didn't know everything from the start (Specialist doctor).

Some managers stated that cooperation both improved and deteriorated and that it was difficult as a manager to support employees.

Cooperation improved at some levels and deteriorated at others. Difficult to support staff in the way that I am used to (First-line manager).

This was stated repeatedly:

The support from colleagues was positive. What was negative was the absence of support and guidance from senior managers (Specialist doctor).

However, the most prominent pattern among the managers' responses was their emphasis on the importance of the positive collaborative culture and a sense of community among healthcare professionals focusing on the needs of patients that emerged during the crisis.

It has been positive, with a sense of community and a hospital spirit (First-line manager).

The interrelatedness mainly concerned the staff on the same hierarchical level, internally between the staff in the units and across units.

### 3.3. Organisational Learning and Knowledge Acquisition

Another common theme was that the crisis had brought about an increased focus on learning and gaining new insight, which according to the respondents had led to a knowledge boost within the organisation. Several managers emphasised that the staff's knowledge had increased significantly during the crisis.

Everyone wants to help, and the staff want to learn new things to provide the best care for patients (First-line manager).

Nurses and doctors emphasised the learning that occurred during the crisis and the importance of having access to specialist skills.

The positive thing has been the help we have received and the fact that colleagues from other units have come to the infection department. It was important that specialist expertise was available and that there was good cooperation with the Intensive Care Unit (ICU) and that the staff at the ICU were available. We have increased our skills in the department, and it has been a steep learning curve (Assistant nurse, ICU).

Some managers also highlighted the complexity of managing the crisis while simultaneously working on learning and knowledge enhancement related to all the new and rapidly changing knowledge about the virus. The focus was not on training new and inexperienced staff but on keeping up with the state of knowledge pertaining to the virus.

It is been hard to work extra and supervise new staff in the department on how to work according to hygiene procedures (Assistant nurse).

The action-oriented discourse present in the material was linked to collegiality and peer learning and associated with efficient problem solving and flexibility.

Our employees have increased their knowledge and have a lot of flexibility (First-line manager).

Positive and negative experiences were reported regarding the enforced flexibility, where staff had to be moved around. Some described having had to perform different tasks than what they were used to, which had been instructive. However, for some nurses, this meant a working environment characterised by considerable uncertainty while caring for the patients.

No training in what I was expected to do. Also had to work in a completely new workplace with completely new tasks, with no training (Specialist nurse).

Organisational resilience was strengthened by the commitment of staff to continually learn and expand their knowledge and to do their utmost to be flexible and adapt to the rapidly changing needs of the organisation.

### 3.4. Information and Communication

A fourth theme in the survey comments concerned access to accurate information. While previous themes largely focused on factors that facilitated resilience, information access and distribution frequently seemed to present barriers to decent work, sustainability, and organisational resilience. Overflow and unclear and ever-changing, contradictory information posed problems. Both insufficient information and information overload were described.

The information about COVID-19 was poorly available –too much and confusing./.../An infinite number of emails (Specialist doctor).

Respondents commented on the communication of information, specifically on how the distribution of information complicated the access to accurate and updated data and also created uncertainty regarding the information given. Several respondents depicted the distribution of information as inadequate. Having to distinguish between outdated and new information was mentioned as a problem.

There was too much unsorted information. When new findings come out, then send out only what is new, not what is new mixed with previous information, as it becomes hopeless to... (Specialist doctor).

The frequency of new information, often contradicting the previous instructions, was raised and linked to the ability to make informed decisions.

A lot of the information circulating at work became contradictory because there was so much new information coming in all the time (caregiver, paediatric nurse, healthcare assistant, etc.).

The reliability of information was also questioned when the right procedure one day could be deemed wrong the next day.

It has been difficult to keep up with certain routines as they could change several times; how to do certain things could be completely wrong in the next work shift (Assistant nurse).

A lot of the information abovementioned referred to hygiene practices and protective equipment; hence, it had bearing on the frontline workers' sense of safety and confidence in their ability to perform their tasks correctly.

Confusing when guidelines changed back and forth and when protective equipment changed (First-line manager).

## 4. Discussion

This study offers a more detailed understanding of the anticipation and learning processes experienced by HCWs during the COVID-19 pandemic [11]. In relation to individually focused scholarship on resilience within nursing management, this article contributes to studies on resilience by focusing on organisational processes and how resilience relates to practise within a specific healthcare organisation. Organisational practices can support and facilitate both individual and organisational resilience by creating favourable work conditions and environments. All the four empirical themes found — problem-solving orientation, cooperation, peer support, and trust, organisational learning and knowledge acquisition, and information and communication — have bearing on organisational resilience. Focusing on the concurrent action, mainly the development and implementation of coping strategies, *resilience capability* is central in managing an ongoing dynamic between different processual practices.

The need for swift action was underlined by the first theme of adopting a *problem-solving orientation.* This theme illustrated the ability to prioritise between tasks, focusing on core activities as being central to resilience capability in a crisis. The respondents' statements revealed that the crisis enabled the organisation to adopt a problem-solving and action-oriented mindset, through which the most pressing matters (care of patients) were prioritised over routines (anchoring work) and democratic decision-making processes. The action-oriented discourse present in the empirical material entailed the rewarding of “doers” and short decision-making processes over administrative work and staff polling. This theme mirrors previous studies linking the prioritisation of patient-related core activities to healthcare organisations' resilience in crisis [17].

The second theme —* cooperation, peer support, and trust* — demonstrated how the crisis brought about cooperation across professional and organisational boundaries, which, in turn, contributed to a better overview for those involved. A sense of community within and across professional and organisational boundaries is described as having facilitated operations and ensured flexibility. Uniting behind a common cause and striving towards a shared goal promoted resilience and constituted a counterforce in the face of exhaustion and mental breakdown of HCWs. A recurring comment was the common desire among staff to help solve problems and together make the best of the situation. Our findings support the previous studies emphasising a supportive culture and social setting to promote resilience and reduce stress and anxiety at work [9, 13–15].

The third theme —* organisational learning and knowledge acquisition *— shows how resilience capability is related to the degree to which an organisation, during a crisis, is receptive to and engages in learning. Gaining knowledge quickly is essential in a crisis where information is constantly being added and updated and the state of knowledge is continuously increasing. When the crisis concerns a new condition, as during COVID-19, it is important that the organisation can absorb and incorporate new knowledge into its operations. The fourth theme illustrates how all themes are interrelated and overlapping. Knowledge acquisition is closely linked to the first theme's emphasis on access to information. Relationships and communication across professional and organisational boundaries, highlighted through the third theme, act as facilitators for knowledge acquisition to take place. This theme ties in with the previous research that has highlighted the ability to assimilate new knowledge and adapt to new circumstances as prerequisites for creating security and predictability in a crisis situation [18].

Finally, the fourth theme, *information and communication*, alludes to the importance for resilience capability of having access to sufficient and accurate, timely information. Rapid decision-making depends on a reliable flow of information, as decisions are made based on all information available at a given time. As rapid decision-making and action constitute prerequisites for operating in a crisis, clarity and timeliness in managing and disseminating information are pivotal for effective operations. Contradictory information and information that recipients could not trust were barriers to the development of resilience. This theme supports and follows up on previous findings demonstrating the importance of employee communication [20–23] and access to information and necessary resources [24–26] to resilience and perceived mandate to take action [27].

In this study, the focus on core activities, i.e., patient care, is brought forward as a facilitator for organisational resilience. This focus implies attention to frontline workers dealing with patients. Resilience becomes dependent on the organisation's ability to perceive and treat frontline staff as critical to successful operations.

The barriers to resilience in this study reflect the results of previous studies that identify adequate information and functioning communication as vital components of a supportive work environment [19, 25, 26].

The study illustrates the interplay between individual and organisational resilience for the ability to manage a crisis [10, 33]. The case articulates how the interrelatedness of individual efforts plays a central role in achieving a resilient organisation. In line with Labrague and Santos [5], this study highlights social support as social networks among colleagues at work as pivotal for an organisation's resilience. Organisational support, however, was not as noticeable. Specifically, noted issues that hindered resilience were poorly communicated information and limited support from management. Issues pertaining to communication and dissemination of information support the previous findings that emphasise functioning communications and clear directives for decent work conditions [28].

In this crisis, collegiality and peer learning appear to have presented an organisational response to the uncertain conditions. This is by no means a given response. An alternative response could have been to rely on routines, protocols, and regulations. The described crisis organisation prioritised core activities while casting aside less urgent and relevant matters. This could be said to constitute a desirable approach also under more regular circumstances. One could argue that certain elements of organising during a crisis should not be confined to crisis management. The view of organising during a crisis can be compared to the shifting perception among organisational scholars of organising during change. The perception of change as a deviating occurrence has given way to a perception of change as the norm.

## 5. Conclusion

The study concludes that COVID-19 resulted in paradoxes, tensions, and new experiences in organisational processes and interactions in healthcare organisations. These create an opportunity for lessons learned, not only in times of crises but also for enabling improved ways of working in accomplishing both acute care and planned care. Furthermore, the conclusions of this study are threefold. First, *resilience capability* is characterised by managing an ongoing dynamic between four types of processual practices: (1) problem-solving orientation; (2) cooperation, peer support, and trust; (3) organisational learning and knowledge acquisition; and (4) information and communication. Second, *resilience capacity* includes three stages of processual practices. Third, *sustainable resilience practices* within nursing management involve a focus on the interaction between the individual, team, and organisational level.

## 6. Limitations and Implications for Nursing Management

By its focus and approach, the article addresses issues of organisational resilience under extraordinary circumstances such as a pandemic and complements the previous literature on nursing management that offer a more individually oriented perspective. A limitation of the study is that the qualitative data are limited to questionnaire statements in response to open-ended questions. The addition of interviews would mean richer material and provide a context useful for interpreting the statements.

Although the conclusion of this case study is transferable to other similar healthcare organisations, further studies in similar contexts would be of interest. More thorough studies of organisational resilience and organisational process practices, including the observations of their relations, could provide a more detailed understanding of the significance of enhancing nursing management during unexpected crises.

The findings of our study illustrate the importance of the collective and the social context for organisational resilience. In line with previous scholars (e.g., [15]), we, therefore, argue that focus should be on the collective responsibility for building a resilient culture rather than on individual leadership. Among our findings' practical implications for nursing management is the call for formal training to achieve resilience among staff [16, 34].

## Figures and Tables

**Table 1:** Descriptive data of the participants in the qualitative study.

	Samples (*N*)	Percentages (%)
*Participants*		
Total	4097	100

*Distribution by gender*		
Female	3377	82
Male	678	17
Prefer not to say/missing	42	1

*Distribution by age*		
≤29	416	10
30–39	889	22
40–49	1016	25
50–59	1151	28
≥60	606	15
Missing	19	<1

*Professional role*		
Registered nurses (including specialist)	1337	33
Physicians (including specialists)	421	10
Assistant nurses (enrolled nurses, child care)	734	18
Healthcare professionals, other physiotherapist, occupational therapist, speech and language therapist, dietician, and biomedical analyst	447	11
Administrative staff, human resources	448	11
Manager	385	9
Other	259	6
Missing	66	2

**Table 2:** Depicting data-derived themes and questionnaire statements.

Themes	Questionnaire statements
Problem-solving orientation	“You had the opportunity to practice the core of your basic profession as the administrative bits were limited.”
	“Positive: quick decisions and execution. Clarity towards the organisation and requirement for action where results were followed up in the near future.”
	“I felt that I became clearer as a manager…easier to be clear when we don't have the luxury of taking into account all the personal nuances of the staff.”
	“Unusually clear objectives for the business. Irrelevant work was pushed aside.”
	“People stopped complaining about the usual, everyone focused on resolving/making the best of the situation.”
	“We worked on what was important, other things were scaled back; lessons for the future!”

Cooperation, peer support, and trust	“There has been a great willingness to collaborate across clinics and within clinics.”
	“Cohesion in the clinic increased.”
	“There was a very good sense of community among all staff and it felt like everyone was 'pitching in' and doing their best.”
	“Good collegial cohesion and cooperation.”

Organisational learning and knowledge acquisition	“What I see as generally positive is increased knowledge and an improved approach to patients with respiratory symptoms.”
	“Increased insight, increased competence, increased understanding”
	“Developed in my professional role and knowledge”
	“Learned a tremendous amount about IVA work in a short time. Grew in my role.”

Information flow and overflow	“Unclear information, different rules, and procedures in different activities.”
	“Often mixed guidelines, protective equipment removed for healthcare workers because “it wasn't needed”-very strange that it then came to be needed anyway.”
	“Negative: the flow of information which was at times unreasonably large, unsynchronised, and rushed as to what applied and for how long.”
	“Lack of reliable information.”
	“Unclear and changing guidelines.”

## Data Availability

Access to data is restricted. However, data are available from the corresponding author upon reasonable request.
